# Effects of prenatal alcohol exposure on the olfactory system development

**DOI:** 10.3389/fncir.2024.1408187

**Published:** 2024-05-15

**Authors:** Fumiaki Imamura

**Affiliations:** Department of Pharmacology, Penn State College of Medicine, Hershey, PA, United States

**Keywords:** Fetal Alcohol Spectrum Disorders, prenatal alcohol exposure, olfactory system, olfactory bulb, development

## Abstract

Fetal Alcohol Spectrum Disorders (FASD), resulting from maternal alcohol consumption during pregnancy, are a prominent non-genetic cause of physical disabilities and brain damage in children. Alongside common symptoms like distinct facial features and neurocognitive deficits, sensory anomalies, including olfactory dysfunction, are frequently noted in FASD-afflicted children. However, the precise mechanisms underpinning the olfactory abnormalities induced by prenatal alcohol exposure (PAE) remain elusive. Utilizing rodents as a model organism with varying timing, duration, dosage, and administration routes of alcohol exposure, prior studies have documented impairments in olfactory system development caused by PAE. Many reported a reduction in the olfactory bulb (OB) volume accompanied by reduced OB neuron counts, suggesting the OB is a brain region vulnerable to PAE. In contrast, no significant olfactory system defects were observed in some studies, though subtle alterations might exist. These findings suggest that the timing, duration, and extent of fetal alcohol exposure can yield diverse effects on olfactory system development. To enhance comprehension of PAE-induced olfactory dysfunctions, this review summarizes key findings from previous research on the olfactory systems of offspring prenatally exposed to alcohol.

## Introduction

Maternal alcohol consumption during pregnancy is the most commonly identifiable non-genetic cause of physical disabilities and damage to the brain in the child. These disabilities or damages are collectively known as Fetal Alcohol Spectrum Disorders (FASD) (Popova et al., 2023). Estimates of the prevalence of FASD in the US and Western Europe range from 0.6 to 5.0% among school-aged children (May et al., 2009, 2014, 2018). There is no known safe amount and timing of alcohol to drink during pregnancy. Some may drink throughout pregnancy, and some may binge drink, consuming a large amount of alcohol in a short period. Human pregnancy is roughly divided into 3 stages known as trimesters of about 3 months each: first trimester – conception to 12 weeks; second trimester – 13 to 27 weeks; third trimester – 28 to 40 weeks. The prevalence of drinking during pregnancy varies by trimester and is higher in the first trimester than in the second and third trimesters (Ethen et al., 2009). According to a 2013 report, approximately 18% of US women consumed alcohol during early pregnancy, and 6.6% binge drank (The NSDUH Report, Substance Abuse and Mental Health Services Administration, 2014). While both binge drinking and chronic low-level drinking during pregnancy are harmful, it is important to note that binge drinking poses a significant risk for serious brain damage (Maier and West, 2001).

There are some common features such as physical features including lower birth weight, shorter stature, smaller head circumference, facial dysmorphism, and neurocognitive deficits including intellectual disability, speech and language delays, poor social skills, and increased risk of anxiety, depression, and ADHD (Riley et al., 2011; Temple et al., 2019). In addition, sensory abnormalities are often observed in children with FASD. They may show signs of being hypersensitive or hyposensitive to the senses of touch, taste, smell, sight, and sound. Particularly, changes in smell/taste sensitivity affect children’s eating behaviors (Carr et al., 2010; Hannigan et al., 2015; Jirikowic et al., 2020). Furthermore, children with a history of heavy alcohol exposure before birth exhibited impaired odor identification (Bower et al., 2013) as well as arhinencephaly (Peiffer et al., 1979). Therefore, it is important to understand how maternal drinking during pregnancy affects the child’s olfactory system. This review summarizes the previous animal studies focusing on the impacts of prenatal alcohol exposure (PAE) on the olfactory system. The author apologizes to those whose work was not included here due to space limitations.

## Studies of prenatal alcohol exposure focusing on the olfactory system

The characteristics of FASD vary in severity and depend on the timing, amount, and pattern of alcohol consumption during pregnancy. Several animal models have been used to simulate maternal drinking episodes. Among them, animal models widely used to see how PAE affects brain development are rodents such as mice and rats (Patten et al., 2014; Almeida et al., 2020). Generally, mice or rats were trained to consume ethanol from their drinking water or diet to simulate chronic drinking during pregnancy. In addition, intraperitoneal injection, subcutaneous injection, and intragastric gavage have been used to simulate binge drinking episodes. As a rough approximation, gestation day (GD) 1–10 of mice and rats corresponds to the first trimester of human pregnancy, GD10-20 (just before delivery) to the second trimester, and postnatal day (P) 1–10 to the third trimester (Almeida et al., 2020). In this review, I adopted a definition of GD0 as the date when the copulation plug was confirmed. When different dates were used in a study, I adjusted the day for a consistent interpretation.

### Development of rodents’ olfactory system

Odors are initially detected by odorant receptors expressed in olfactory sensory neurons (OSNs) within the olfactory epithelium (OE). These OSNs extend their axons to the glomeruli of the olfactory bulb (OB) to form synapses with mitral and tufted cells, which serve as OB projection neurons transmitting olfactory information to the olfactory cortex. In the OB, the activity of mitral/tufted cells is modulated by OB interneurons such as periglomerular cells and granule cells, which synapse with dendrites of mitral/tufted cells within the glomerular layer (GL) and external plexiform layer (EPL), respectively.

The development of the rodents’ olfactory system has been studied and summarized in detail in other studies (Treloar et al., 2010; Kim et al., 2023). Briefly, the OE is generated from the olfactory placodes, a thickened ectoderm in the head region. In mice, the olfactory pits begin invaginate from the olfactory placode around GD10. The nostrils are narrowed to small slits and the olfactory pit has further invaginated into a more complex nasal cavity by GD11.5 (Miller et al., 2010b). The invaginated olfactory pits differentiate into OE where OSNs are generated. Generation of OSNs in the OE begins around GD11 and turns over throughout life (Eerdunfu et al., 2017; Nguyen and Imamura, 2019). On the other hand, the OB is located at the most anterior region of the brain in rodents. In mice, the formation of the OB begins with the evagination of the anterior end of the telencephalic vesicle around GD11 (Miller et al., 2010b; Imamura et al., 2011). Mitral/tufted cells are generated from radial glial cells in this developing OB between GD9 and GD17; while mitral cells are mostly generated between embryonic day GD9 and GD13, peaking at GD11, tufted cells are born later, between GD12 and GD17 (Hinds, 1972; Blanchart et al., 2006; Imamura et al., 2011; Hirata et al., 2019). OB interneurons are mostly generated during late gestation and early postnatal stages and are continuously newly born throughout life (Hinds, 1968; Imayoshi et al., 2008).

OSN axons first reach the developing OB at GD11 and penetrate the basement membrane to form an olfactory nerve layer by GD12 (Miller et al., 2010a). The immature mitral/tufted cells have multiple broadly spread apical dendrites, and they begin to form protoglomeruli with OSN axons around GD15 (Treloar et al., 1999; Blanchart et al., 2006). Synapse formation in the OB also starts at this stage in the GL, followed by the EPL and granule cell layer (GCL) (Hinds and Hinds, 1976). Dendritic refinements of mitral/tufted cells, such as discrimination of primary and secondary dendrites and retraction of supernumerary primary dendrites, occur during early postnatal days (Lin et al., 2000; Aihara et al., 2021). Axonogenesis of mitral/atrial cells begins around GD11.5 immediately after final differentiation, and they extend between GD12 and GD14 to form the lateral olfactory tract (Lopez-Mascaraque et al., 1996; Walz et al., 2006). Axons of mitral/tufted cells target the piriform cortex, anterior olfactory nucleus, olfactory tubercle, amygdaloid cortex, and entorhinal cortex, consisting of the olfactory cortex. Many neurons in the olfactory cortex are born during similar stages with the mitral/tufted cells, GD11 – GD18 (Martin-Lopez et al., 2017, 2019; Aerts and Seuntjens, 2021).

### Effects of PAE on the rodent olfactory system

Different timing in ethanol exposure may cause different effects on the olfactory system development. Many rodent studies simulated exposure to alcohol during pregnancy and the findings are summarized in Table 1. A study fed pregnant mice with 10% EtOH in drinking water throughout pregnancy (Akers et al., 2011). In this case, P60 offspring exhibited the greatest volume reduction in the OB among 62 brain regions examined with MRI and showed impaired discrimination between similar odors (80% R-carvone/20% S-carvone vs. 20% R-carvone/80% S-carvone) but left odor memory intact (Akers et al., 2011). Similarly, when pregnant female rats were fed with a 35% ethanol-derived calorie (EDC) liquid diet from GD6 to GD20 of pregnancy, offspring showed a volume decrease in the OB at P3 (Barron and Riley, 1992). Interestingly, the P3 rats born from females fed with 35% EDC did not show a preference for the odor paired with milk infusion, and the P10 rats did not avoid the odor associated with lithium chloride injection, which induced a mild toxicosis (Barron et al., 1988). However, the P100 adult rats born from females fed with 35% EDC showed the same level of odor aversion learning as the control group (Barron et al., 1988). Another study fed pregnant mice and pups with a 20% EDC liquid diet from GD13 to P21, equivalent to humans’ second and third trimesters and early postnatal weeks (Nyouist-Battie and Gochee, 1985). This study showed an approximately 25% volume reduction in the OB of ethanol-fed mice at P21 compared to a normal diet-fed group, with reductions in the volume of the GL, EPL, and GCL, while the laminar organization and cellular cytoarchitecture were not substantially altered by ethanol.

**Table 1:** Effects of prenatal alcohol exposure on the rodent olfactory system.

Species Timing / Duration	Route (dose)	Age examined	Effects on the olfactory system	References
Mice GD0 – GD19	Drinking water (10%)	P60	Decrease in OB volume Failed to discriminate relatively similar odors	Akers et al. (2011)
Rats GD0 – GD19	Intragastric gavage (6.0 g/kg daily; 22.5% solution)	GD20 & P10	Decrease in OB volume Reduction in the granule cell numbers	Maier et al. (1999)
Rats GD6 – GD20	EDC liquid diet (35%)	P3	Decrease in OB volume; no odor preference paired with milk	Barron et al. (1988) and Barron and Riley (1992)
		P10	no odor aversion associated with mild toxicosis	
		P100	the same level of odor aversion associated with mild toxicosis as the control group	
Mice GD7 or GD8	Intraperitoneal injection (2.9 g/kg- 25% solution or 2.8 g/kg- 23.7% solution; twice at four-hour intervals)	GD17	Decrease in OB volume	Parnell et al. (2009), Godin et al. (2010), and Lipinski et al. (2012)
GD8.5			Increase in OB volume	
Mice GD7 – GD11	Liquid diet containing ethanol (4.8%)	GD17	Decrease in the length of the right OB	Parnell et al. (2014)
GD12 – GD16			No significant defects	
Rats GD11 – GD20	EDC liquid diet (35%)	P40 - P48	Gene expression changes in OB * Enhanced ethanol intake at P15	Youngentob et al. (2007a), Youngentob et al. (2007b), Middleton et al. (2009), Youngentob and Glendinning (2009), and Gano et al. (2020)
Mice GD13 – P21	EDC liquid diet (20%)	P21	Decrease in OB volume	Nyouist-Battie and Gochee (1985)
Rats P4 – P9	Intragastric gavage (4.5 g/kg daily; 5.1% or 10.2% solution)	P10 & adult (> P90)	Decrease in OB volume Reduction in the granule and mitral cell numbers	Bonthius and West (1991) and Bonthius et al. (1992)
	Intragastric gavage (6.6 g/kg daily; 2.5% solution)		No significant reduction in the granule and mitral cell numbers	
Mice P4 – P9	Intraperitoneal injection (4.4 g/kg daily; 20% solution)	Adult (P110 – P122)	Decrease in OB volume Reduction in the granule cell numbers	Todd et al. (2018)
Mice P7	Subcutaneous injection (2.5 g/kg- 20%; twice at two-hour intervals)	3-month-old	Enhanced odor-evoked local field potential in the OB and anterior piriform cortex	Wilson et al. (2011)

In another study, alcohol was administered by intragastric gavage (6.0 g/kg/day) to pregnant rats from GD0 to GD19, which corresponds to the first two trimesters of human pregnancy (Maier et al., 1999). Compared to the control group that received an isocaloric maltose-dextrin solution, the offspring of ethanol-fed females had smaller OBs with a reduced number of granule cells at GD20 and P10. Exposure to alcohol during the third trimester also affected OB formation. Rat pups were reared artificially and were administered alcohol with intragastric gavage (4.5 g/kg daily; administered either as a 5.1% or 10.2% solution) over P4 through P9. This alcohol exposure paradigm also reduced the OB volume and caused the reduction of the number of granule cells as well as mitral cells in P10 and adult (> P90) OBs (Bonthius and West, 1991; Bonthius et al., 1992). Interestingly, a higher daily dose (6.6 g/kg) but administered continuously with a lower (2.5%) ethanol concentration did not affect the number of either granule or mitral cells (Bonthius and West, 1991). Decreases in OB volume and number of granule cells were also observed in adult mice that received intraperitoneal injections of ethanol (4.4 g/kg) daily over P4 to P9, but not at lower doses (2.2 g/kg) (Todd et al., 2018). Therefore, chronic PAE impairs the OB formation and affects the generation and survival of OB neurons. Although the underlying mechanisms of OB damages are not known, gene expression profiling revealed a PAE, feeding with 35% EDC from GD11 to GD20, affected the expression of genes involved in neuronal development, synaptic transmission, and plasticity as well as inflammatory-related genes during adolescence (P40–P48) (Middleton et al., 2009; Gano et al., 2020). Moreover, the rats exposed to gestational ethanol showed enhanced ethanol intake as well as different sniffing responses to ethanol odor at P15, but the ethanol preference was absent at P90 (Youngentob et al., 2007a,b; Youngentob and Glendinning, 2009).

In addition to chronic PAE, acute PAE caused by binge drinking also affects the development of the olfactory system. To cause an acute PAE, several studies used the intraperitoneal ethanol injection method. When ethanol (2.9 g/kg) was administered intraperitoneally to pregnant female mice twice (four-hour intervals) at GD7, MRI measurement at GD17 found a reduction in overall brain size with marked volume reduction in the OB (Godin et al., 2010; Lipinski et al., 2012). Reduction of OB volume at GD17 was also observed with the intraperitoneal ethanol exposure at GD8 (2.8 g/kg; twice at four-hour intervals) (Parnell et al., 2009), while the same ethanol exposing paradigm performed at GD8.5 caused approximately 10% increase of the OB volume (Lipinski et al., 2012). Since some of the mice that showed a reduction of OB volume also had abnormal nasal cavity, defects in the development of olfactory sensory neurons might affect the OB formation in these mice (Parnell et al., 2009; Godin et al., 2010). On the other hand, the same group fed the pregnant mice with the 4.8% (v/v) ethanol-containing liquid diet for five days, from GD7 to 11 and from GD12 to 16 (Parnell et al., 2014). In this case, GD 7–11 and GD 12–16 ethanol-exposed groups showed a significant decrease in the volumes of the cerebellum and hippocampus at GD17, respectively, but no significant change in OB size was observed except for a shortening of the right OB of mice exposed to ethanol from GD7 to GD11.

Another study simulated binge drinking in the third trimester by causing acute PAE with subcutaneous injection of ethanol (2.5 g/kg; twice at two-hour intervals) into P7 mouse pups (Wilson et al., 2011). This treatment caused widespread cell death within 1 day of exposure, with the highest levels in the neocortex, intermediate levels in the dorsal hippocampus, and relatively low levels in the primary olfactory system including OB and piriform cortex. The acute PAE did not change the odor investigation or odor habituation in 3-month-old mice compared to saline-administered controls, whereas the hippocampal-dependent object place memory was significantly impaired. Interestingly, odor-evoked local field potential activity was enhanced in the OB, anterior piriform cortex, and hippocampus. These data suggest that the activity of neural circuits involved in odor information processing can be modified by acute PAE at a later gestational stage, which may contribute to specific behavioral abnormalities seen in children with FASD.

These results from rodent studies indicate that timing, quantity, and style of drinking are important to understanding the impact of PAE on olfactory system development. A previous study showed that acute PAE induced by intraperitoneal ethanol injection (2.9 g/kg) at GD11, but not at GD6, caused apparent deficits in the social behavior of male rat offspring; reduction of social investigation, contact behavior, and play fighting (Mooney and Varlinskaya, 2011). Considering the pivotal role of the olfactory system in rodent social behavior (Bakker et al., 2022), it is plausible that PAE-induced defects in olfactory information processing resulted in impaired social behavior.

### Other animal models of PAE

Several other studies used non-rodent animals to examine the effects of PAE on the development of OB. For example, pregnant sheep were administered with alcohol. A moderate dose of alcohol was infused intravenously (1.75 g/kg) on 3 consecutive days followed by 4 days without alcohol beginning on GD 4 and continuing until GD 132, which corresponds with the end of the third trimester of human pregnancy (Washburn et al., 2015). In contrast to the findings from rat studies (Bonthius and West, 1991; Bonthius et al., 1992), there was no change in the number, density, or volume of mitral cells in the fetal (GD133) sheep OB, although it does not exclude the presence of functional abnormalities or the reduction in number of granule cells. In another study, fewer actively proliferating cells were found in the OBs of newborn monkeys born from females who voluntarily consumed alcohol (a maximum of 3.5 g alcohol/kg body weight on 4 days of the week) starting in the mid-gestation stage (Burke et al., 2016).

## Discussion

As summarized in this review, it’s evident that the olfactory system is vulnerable both to chronic and acute PAE. In particular, the reduction in OB volume was prominent and was often associated with a decrease in the number of granule cells and mitral cells, suggesting that PAE affects the neurogenesis of OB neurons. This view is also supported by studies in animal models and human patients showing that PAE reduced the proliferation of neural stem cells in the subventricular zone (SVZ) (Roitbak et al., 2011; Dong et al., 2014; Marguet et al., 2020). Moreover, defects in adult neurogenesis may also contribute to PAE-induced reduction of OB volume and olfactory function in adult rodents, as new OB interneurons are continuously produced in the SVZ of the adult rodent brain (Whitman and Greer, 2009).

The impairment in the olfactory system development likely leads to abnormal olfactory information processing. This, in turn, may contribute to abnormal smell sensitivity and impaired odor identification seen in children with FASD. However, studies to date have varied in terms of timing, duration, dosage, and route of ethanol administration as well as the age of offspring investigated, making it still challenging to formulate a cohesive understanding of how PAE precisely influences the child’s olfactory system. Systematic identification of differences in the effects of PAE at different stages of olfactory system development may provide valuable insights into important windows of vulnerability. In addition, it is necessary to study in more detail the effects of PAE on the structure and function of regions involved in olfactory processing other than the OB, such as olfactory epithelium and olfactory cortex.

Furthermore, while diverse *in vivo* and *in vitro* studies have elucidated various signaling pathways affected by PAE during brain development (Hashimoto-Torii et al., 2011; Mohammad et al., 2020; Fischer et al., 2021; Salem et al., 2021; Sambo et al., 2022), this type of research has so far been insufficient for the olfactory system. Understanding the molecules and pathways affected by PAE in the developing olfactory system could shed light on potential mechanisms underlying the etiology of abnormal sense of smell. Integrating findings from diverse experimental models and methodologies could facilitate the construction of comprehensive models that capture the multifaceted nature of PAE-induced alterations in olfactory system development and could inform targeted intervention strategies aimed at mitigating the detrimental effects of PAE on olfactory function. Therefore, collaborative efforts across disciplines, including neuroscience, developmental biology, and clinical research, are essential to surmount the complexities associated with understanding and addressing the consequences of PAE on olfactory function and beyond.

## Author contributions

FI: Writing – original draft, Writing – review & editing.
